# *Helicobacter pylori* Infection in a Pediatric Population from Romania: Risk Factors, Clinical and Endoscopic Features and Treatment Compliance

**DOI:** 10.3390/jcm11092432

**Published:** 2022-04-26

**Authors:** Oana-Maria Rosu, Nicoleta Gimiga, Gabriela Stefanescu, Carmen Anton, Gabriela Paduraru, Elena Tataranu, Gheorghe G. Balan, Smaranda Diaconescu

**Affiliations:** 1 Doctoral School, George Emil Palade University of Medicine, Pharmacy, Science, and Technology of Targu Mures, 38 Gheorghe Marinescu Str., 540139 Targu Mures, Romania; oana7772@yahoo.com; 2 Clinical Department of Pediatric Gastroenterology, “St. Mary” Emergency Children’s Hospital, 700309 Iasi, Romania; gabyspulber@gmail.com; 3 Faculty of Medicine, “Grigore T. Popa” University of Medicine and Pharmacy, 16 Universitatii Str., 700115 Iasi, Romania; carmen_ro2008@yahoo.com (C.A.); balan.gheo@yahoo.com (G.G.B.); 4 Gastroenterology and Hepatology Institute, “St. Spiridon” Emergency Hospital, 1-3 Independetei Str., 700115 Iasi, Romania; 5 Clinical Department of Pediatrics, Sf. Ioan cel Nou, Emergency Hospital, 720224 Suceava, Romania; elena8025p@yahoo.com; 6 Faculty of Medicine, “Titu Maiorescu” University of Medicine, 67A Gheorghe Petrascu Str., 031593 Bucharest, Romania; turti23@yahoo.com

**Keywords:** *Helicobacter pylori*, children, endoscopic, treatment compliance

## Abstract

Background and Objectives: The aim of this study was to investigate the association between *H. pylori* positivity with specific symptoms, risk factors and endoscopic patterns among the pediatric population in northeastern Romania. Materials and Methods: A prospective study was performed in 18 months on 185 children aged 6–18 years with an indication for upper digestive endoscopy. Demographic, anamnestic, symptomatic, endoscopic and histologic data were recorded. Results: Of 116 *H. pylori*-positive children, the most affected group was 15–17 years. Most (65.5%) of them were girls, with a significant association (*p* < 0.001). The majority (66.4%) lived in a rural area and 47.4% of children had an unsafe source of water and lived in overcrowded houses with no basic sanitary comfort. The most frequent symptom was epigastric pain (56.9%), and the main endoscopic appearance was nodularity and hyperemia. Patients diagnosed with *H. pylori* started triple-therapy treatment for 14 days. Only 13.8% stopped the treatment, mainly because of a misunderstanding of the treatment protocol (9.5%). Conclusions: Romanian teen girls living in rural areas are at high risk for *H. pylori* infection. Epigastric pain and endoscopic nodularity of the gastric mucosa were associated with the infection. As the resistance of the bacteria is unknown in our country, future research is needed in order to improve the eradication rate.

## 1. Introduction

*Helicobacter pylori* infection is one of the most prevalent in the world. The infection occurs during childhood and can be carried throughout life if left untreated. Epidemiologically, *H. pylori* infection can be characterized by a linear increase with age in Western industrialized countries and an increased number of infected children in developing countries [1].

The Global Health Epidemiology Research Group conducted a 198-article meta-analysis, concluding that *H. pylori* infection among children and adolescents globally still has a fairly high prevalence [2].

The interest in *H. pylori* infection among the pediatric population has been growing in recent years, and gastric disorders due to infectious disease have led to studies in this direction. This association was confirmed for children in several studies, but these studies included a limited number of cases.

As shown by many epidemiological studies, the epidemiology of *H. pylori* in children is constantly changing. Gastric infection with *H. pylori* is influenced by factors of the host’s gastric mucosa, environmental factors and bacterial virulence [3].

In Romania, there is a lack of studies on the prevalence of *H. pylori* infection in children. However, there are a few research studies on some regional areas. In northwestern Romania, the prevalence of dyspepsia among children rises to 40%, according to a study from 2002 conducted on 267 patients with an age range of 5 to 18 years old [4]. A more recent study from 2018, which enrolled 7100 pediatric patients from Cluj-Napoca, Romania, also reported a 25% prevalence [5].

In our country, there are important risk factors, mainly in rural areas, namely, the type of housing that influences the sanitation conditions, low socioeconomic status, a large number of family members, sleeping in common beds and the deficiencies of the water supply network. At the same time, only 29.9% of children in Romania are fed exclusively with breast milk in the first months of life, which could be an additional risk factor, if we take into consideration its possible protective factor against *H. pylori* [6].

According to the European Society of Pediatric Gastroenterology and Nutrition (ESPGHAN)/North American Society of Pediatric Gastroenterology and Nutrition (NASPGHAN) guidelines, *H. pylori* diagnostics in the pediatric population require a positive culture or a histopathologic finding of *H. pylori* gastritis, accompanied by one more examination, such as a urease rapid test. In addition, the treatment of pediatric *H. pylori* infection is based on updated protocols of the ESPGHAN/NASPGHAN guidelines, revised in 2016. The first-line treatment should be administered according to the antimicrobial susceptibility of the *H. pylori* strain, and if not determined, the guidelines recommend adapting the treatment according to the regional antimicrobial resistance trend or the eradication prescription which is more efficient locally. Clarithromycin resistance is higher than 15%, so triple therapy should not include this antibiotic. In addition, sequential therapy should be avoided in children [7]. Eradication control must be performed preferably by a non-invasive test (urea breath test in children over 6 years or the detection of faecal antigen by ELISA method with monoclonal antibodies) 4–8 weeks after the end of the antibacterial treatment [7,8,9]. The leading causes of treatment failure are side effects, reduced compliance and antibiotic resistance.

The standard triple therapy based on clarithromycin, previously recommended [10], is widely prescribed in our country, especially in the primary care network. Alternative schemes are recommended only in tertiary centers. Romania holds a leading position in the European Union in terms of over-the-counter use of antibiotics, estimated at 30% [11]. The widespread use of antibiotics (clarithromycin for respiratory infections) in the general population has led to an increase in the rate of antibiotic resistance at rates between 49.2% in Spain and 0.8% in the Netherlands [12]. In Romania, there are currently no studies on antibiotic resistance of *H. pylori* in children.

This study proposes an investigation of the association between *H. pylori* positivity with symptoms, risk factors and endoscopic patterns among the pediatric population in northeastern Romania. In addition, we hereby present an assessment of the socioeconomic and demographic factors influencing infection and treatment adherence.

## 2. Materials and Methods

A prospective study consisting of 185 patients, aged 6–17 years, admitted to the Pediatric Gastroenterology Unit of St. Mary Children’s Hospital, Jassy, Romania, who underwent upper digestive endoscopy due to various gastrointestinal complaints was conducted between February 2019 and July 2020. The presence of *H. pylori* infection was diagnosed by histopathology and rapid urease testing.

Patients who were aged less than 6 years or aged over 18 years or who received a proton pump inhibitor, antibiotics or antibacterial therapy four weeks before endoscopy, and patients with other conditions such as ulcerative colitis, Crohn’s disease or celiac disease, were excluded.

The study was approved by decision no. 4363/20 February 2019 of the Research and Ethics committee of ”St. Mary” Hospital. All patients included in the study agreed to participate by approval and signature of informed consent by parents or legal guardians and underwent upper digestive endoscopy with gastric biopsy for *H. pylori* testing.

After endoscopy, the *H. pylori*-positive patients were treated according to ESPGHAN guidelines, with doses depending on bodyweight. For patients weighing 15–24 kg, PPI 40 mg, amoxicillin 500 mg and metronidazole 500 mg divided in 2 doses was prescribed. For patients weighing 25–34 kg, PPI was prescribed 30 mg twice daily, along with amoxicillin 500 mg twice a day and metronidazole 500 mg in the morning and 250 mg in the evening. For pediatric patients over 35 kg, the following was recommended: 40 mg of PPI, 1000 mg of amoxicillin twice daily and 500 mg of metronidazole twice daily.

A simple closed-ended questionnaire was completed by telephone, and personal data was protected to maintain the patient’s anonymity; it was completed after the completion of the treatment. The purpose was to collect information about the pediatric patient participating in the study, the type of home toilet, the sources of drinking water from home, the period of breastfeeding, the number of people in their household, family history of upper digestive pathology, history of antibiotic therapy and side effects of the treatment. All children and their families were also questioned about gastrointestinal symptoms or complaints: epigastric pain, recurrent abdominal pain and vomiting.

In addition, the study participants were asked about the recommended treatment schedule, if it was administered correctly and completely, and if not, what the causes that led to the discontinuation of treatment were.

Statistical analysis: all data was processed (coding, entry, validation and analysis) with IBM SPSS Statistics for Windows, v20.0 (Armonk, NY, USA). Depending on the type of variable, we used Pearson’s chi-squared test and calculated Lambda (for association or asymmetry). Categorical variables are expressed as numbers and/or percentages. All statistical tests are 2-tailed and have a reported *p*-value < 0.05, which was considered statistically significant for the study, as in all former studies where *p*-values less than 0.05 or near 0.05 were considered of statistical significance.

## 3. Results

### 3.1. Demographic Characteristics of the Study Population

The study included 185 children aged 6–17 years admitted in the gastroenterology department for various gastrointestinal complaints who underwent upper digestive endoscopy with gastric biopsies for *H. pylori.*

The children were divided into groups by age, respectively: 6–9 years range, 10–14 years range and 15–17 years range. The mean age of the children was 13.12 years with a standard deviation of 3.27 years. The 15–17 years age group included most children, followed by the 10–14 age group. The majority, 126 (68.10%), were girls and only 59 (31.90%) were boys. Most children, 116 (62.70)%, were living in a rural area, and only 69 (37.30%) were living in an urban area. The children in the sample were breastfed for more than 6 months at a rate of 61.62% (114 children). Table 1 summarizes the study population characteristics.

**Figure 1:**
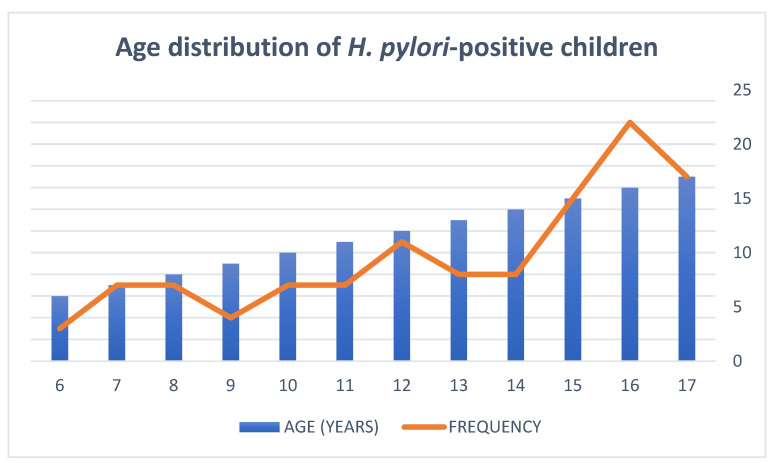
Age distribution of *H. pylori*-positive children.

Most of the *H. pylori*-positive diagnoses in our study were found amongst children older than 15 years old, from rural areas., The prevalence of the *H. pylori* infection was found to progressively rise with age. (Figure 1).

### 3.2. Risk Factors in Pediatric Helicobacter Pylori Infection

Risk factors are important to correlate with risks of infection. As such, we investigated some of the possible ambient and family factors that could enhance the development of *H. pylori* infection. These factors regard hygiene routine and sanitary conditions at home, number of people inhabiting the house, family history of dispeptic disorders, breastfeeding and pets present in the household.

Nearly half (47.4%) of infected children had improper or unsafe water sources and an outdoor toilet, and 14% had overcrowded families. Infected children were breastfeed less than 6 months at a rate of 42.2%. Only 24.9% of the sample owned pets, with no statistical significance in the study group.

Family history of gastric pathology was identified in only 14.7% of cases. Other associatted risk factors may be previous respiratory infections; 77.5% had a history of 1–2 infections/year (*p* < 0.001). The history of antibiotics previously received for various other pathologies in the case of enrolled patients was extremely varied in preparations and combinations alike. The statistical analysis showed that the main category of patients was treated with clarithromycin and amoxicillin and clavulanic acid (41.3%), as well as clarithromycin monotherapy (32.7%) (Table 2).

### 3.3. Clinical Characteristics and Endoscopic Patterns

Gastrointestinal complaints were reported by 56.8% of patients with epigastric pain, followed by recurrent abdominal pain (14.6%) and subsequently by the concomitant presence of epigastric pain and vomiting (12.9%).

Endoscopic aspects were mainly hyperemic lesions (37.8%) and nodular lesions (20.5%) followed by the association between nodular and hyperemic lesions (19.4%) (Table 3).

The Sydney System is used internationally to classify chronic gastritis. This system can provide information on the density, activity, chronic inflammation, atrophy and intestinal metaplasia of *H. pylori*. The diagnostic criteria include confirmed gastritis according to the updated Sydney classification and at least one positive test, such as RUT, molecular tests where available, polymerase chain reaction (PCR) or in situ fluorescent hybridization [7].

The Sydney System semi-quantitatively grades the density of *H. pylori* infection on a scoale from zero to three (none, mild, moderate and marked). On the histological section, *H. pylori* can be recognized as a short, curved or spiral bacillus on the epithelial surface or in the mucus layer. We observed in our study that there is a relationship between chronic inflammation and *H. pylori* (*p* = 0.002) (Table 4).

### 3.4. Treatment Regimens and Compliance

Most of the *Helicobacter pylori*-positive patients were treated with two different regimens: 1—amoxicillin, clarithromycin and a selected proton pump inhibitor (PPI) for a period of 14 days (42.2%) or 2—amoxicillin, metronidazole and a PPI for 14 days (53.4%). Only 13.8% of patients stopped the treatment, and the main cause that led to the discontinuation of treatment was the misunderstanding of the treatment protocol (9.5%) or the presence of side effects (4.3%).

Epigastric pain was the persisting symptom in 27.5% of patients, and side effects such as nausea, vomiting or diarrhea were present in 29.3% of cases, with no differences in treatment regimen. (Table 5).

The method of determining eradication was used according to ESPGHAN guidelines. At least four weeks after completing triple-*H. pylori* infection eradication therapy, a non-invasive test such as a faecal antigen test was performed. Of all the 116 positive patients, only 43 of them participated in the follow-up, with a negative faecal antigen test (37%).

## 4. Discussion

*Helicobacter pylori* (*H. pylori*) is a gram-negative type bacterium, currently known as the source of the most common infection within all age groups and is a challenging health issue [13,14,15,16]. *Helicobacter pylori* is often transmitted in childhood from saliva by person-to-person interaction [17]. As such, intra-familial routines have the most important role. The spread of the bacteria can also be caused by contamination of food or drinking water. Increased prevalence of infection can be caused by untreated water, overcrowded housing or poor hygiene [10,18].

According to literature and current studies, infection prevalence in Sweden was 13.6% in children aged 18 to 24 months, while in Ireland and Germany the rates of *H. pylori* infection were 8.6% and 2.4%, respectively. Bulgaria is the country with the most *H. pylori*-infected children aged between 1 and 17 years, while the lowest infection rate was reported in the Netherlands [19].

According to our current knowledge, the present study is the first to investigate pediatric infection with *H. pylori* by endoscopic aspects, treatment compliance and risk factors.

Colonization with *H. pylori* starts early in life [20]. As age increases, so does exposure to various sources of infection, increasing the rate of infection. This could be an explanation for the higher rate of infection among schooling children. In our study, there was a progressive increase in the prevalence of *H. pylori*. The infection rate may be higher for children who attend poorly sanitized schools, who have an outdoor toilet or who do not have clean drinking water [21]. In Romania, the chances of transmitting the infection from person to person increase because there are many overcrowded schools, and the transmission can take place at home too, as long as there is an infected family member [21].

In our study, the prevalence rate was 62.7% among symptomatic children. Corojan et al. summarize studies from the literature on the prevalence of *H. pylori* infection among children, which has increased since 2003 when it was 20%, reaching 25% in 2018 in northwestern Romania. However, the main measure was the result of serological tests for *H. pylori* antibodies and not gastric biopsies. The authors also found that over 40% of adult patients in Romania have or had an *H. pylori* infection. However, similar to the other authors, the infection rate among the pediatric population was found to be constantly increasing [22].

Non-invasive testing for the initial diagnosis of *H. pylori* in children is not recommended. In addition, the eradication scheme is not recommended to be used after such a test. Our study used diagnostic methods among those recommended by international guidelines [7]. To rule out possible false-negative results, we used previous use of PPIs and antibiotics as exclusion criteria. Prior use of PPIs may give false-negative results of invasive diagnostic tests for *H. pylori* infection due to suppression of replication. In addition, previous use of antibiotics can suppress the growth of bacteria. It can lead to false-negative results in all recommended diagnostic tests, except serology, which is not recommended as a diagnostic method in children.

Biopsies were taken according to the recommendations of current guidelines. These recommendations suggest that at least six biopsies are required. Of these, two from the antrum and two from the corpus should be taken for histopathology. A biopsy of the antrum and one of the corpus are harvested if bacterial culture is available. At least one biopsy is collected for additional diagnostic tests such as RUT [7]. In addition, we took biopsies from the areas with the most suggestive changes in the gastric mucosa. All these biopsies with negative results on histopathology and RUT, to which we add the exclusion criteria, make false-negative results unlikely. Skrebinska et al. conducted a study based on the diagnosis of *H. pylori* infection on serology and histopathology. The conclusion they reached is that the discrepancy was due to false positive serology rather than false negative histology [23]. The molecular diagnosis at the time of this study was not possible in our unit.

Our study confirms a difference of statistical significance in the incidence of *H. pylori* infection in boys vs. girls (65.5% vs. 34.5%, *p* < 0.001). The explanation for this difference may be the fact that girls may be more thoughtful about the symptoms they feel and may have a higher predisposition to digestive symptoms, thus leading to greater addressability by medical services. However, there was a slightly higher infection rate in boys (67.7%) compared to girls (60.3%), with no statistically significant difference. In contrast, the meta-analysis by Martel and Parsonnet concluded that there was no significant relationship between sex and the incidence of *H. pylori* infection [24].

According to the ESPGHAN/NASPGHAN guidelines, the acquisition of gastric infection with *H. pylori* takes place around the age of 10 years, and most of the patients have fairly long asymptomatic periods [7]. Domsa et al. have conducted studies that have seen an increase in the frequency of infection as the age of the patients studied increases [5]. The Global Health Epidemiology Research Group found that the prevalence of *H. pylori* infection was higher in older children than in younger children, with the highest percentage being in the 13–18 years age group (41.6%) [2]. Similarly, in our study, the highest incidence was in the 15–17 years group.

Compared to developed countries, the risk factors for *H. pylori* infection are higher in developing countries. These risk factors include poor socio-economic conditions, poor hygiene, overcrowding families and living with an infected family member [25]. Similarly, we found in our study that out of total number of patients, 14.1% had overcrowded housing and 43.2% consumed fountain water. Of the 116 *H. pylori*-positive children, 14.7% had overcrowded housing and 47.4% of them consumed well water, due to the high addressability of patients from rural areas (62.7% of the total number of study participants).

Recent studies in the Japanese literature show a link between *H. pylori* positivity and daily contact with dogs. Other studies suggest that they found the same strains in two dogs and their owner [26]. In the studied population, the owners of pets consisted of 21.6% of the sample, of which 8.1% had dogs, a finding which was not statistically significant.

After researching several studies, Soltani et al. found a significant correlation between *H. pylori* infection and breastfeeding. The results are inconclusive, as some studies showed a positive correlation between breastfeeding and risk of infection while other studies revealed that breastfeeding could be a protective factor against *H. pylori* infection. In our study, positive and negative *H. pylori* children were all breastfed for more than 6 months, therefore, large and long-term clinical observations are further needed for a strong conclusion regarding this correlation [27].

It is recommended that *H. pylori* infection be investigated among vulnerable groups such as family members inhabiting the same residence as positive *H. pylori* patients, persons with a family history of dyspeptic disease or a disease associated with *H. pylori* infection (e.g., active duodenal ulcer). The guidelines and protocols agree on the fact that, once *H. pylori* infection is identified, therapeutic intervention is mandatory unless there are other medical reasons for not doing so [28].

Of the 185 patients studied, only 31 (16.8%) reported a family history of gastric pathology, of which 17 (14.7%) were related to positive *H. pylori* patients and 14 (20.3%) were related to negative *H. pylori* patients. The diagnoses declared by the family members were: a case of gastric cancer in the father of a patient, 23 cases of peptic ulcer disease, 17 of which were *H. pylori*-positive and 7 cases of chronic gastritis of which five were *H. pylori*-positive. There was no significant statistical correlation between *H. pylori* infection and family history of upper digestive pathology (*p* = 0.32). Few parents reported the history and there is no possibility to verify the accuracy of the information received.

In Romania, treatment regimens for respiratory infections in children widely include clarithromycin [29]. Thus, a trend in the history of antibiotic treatment including clarithromycin monotherapy or clarithromycin with amoxicillin and clavulanic acid can be observed in our study, too.

The clinical manifestations are non-specific, and some may indicate the presence of complications. In the study by Correa Silva et al., there was no positive correlation between gastrointestinal symptoms, pain, pain features and *H. pylori* infection but there were some associations with nausea [30]. In addition, in the meta-analysis by Spee et al., no correlation was found between clinical features and infection, other than epigastric pain [31]. Other symptoms such as persistent vomiting, gastrointestinal bleeding, iron deficiency anaemia (ADI) of unspecified cause and malnutrition may be caused by comorbidities or complications of the infection and require further investigations in order to eliminate confusion factors and conduct proper therapy [32].

In Kirdy et al.’s study, the main indication for upper gastrointestinal endoscopy investigation was abdominal pain. They found that there was a statistically significant association between epigastric pain and *H. pylori* infection [33], which was also reported by Yang et al. and Ng et al. [34,35]. Consistent with these research studies, we found that the main symptom was epigastric pain, both in the *H. pylori*-positive group (56.9%) and in *H. pylori*-negative patients (56.5%). Moreover, compared to *H. pylori*-negative patients, the patients with *H. pylori* infection had associated vomiting (13.8%) or had recurrent abdominal pain (15.5%).

Nodular endoscopic appearance is one of the signs of *H. pylori*-associated gastritis and may be associated with a severe gastritis in children [36]. Tatevik et al. found in their data that, in the *H. pylori*-positive group, the most frequent endoscopic lesions were the erosive ones, and the nodular lesions were found exclusively within this group [37].

A report by Toyoshima et al. associated *H. pylori* infection with the nodular appearance of the cardia, considering this association a more accurate in diagnosis. In addition, the sensitivity of the nodular endoscopic aspect in the cardia was significantly higher than in the antral area. This report concluded that cardia nodularity may be a predictor of *H. pylori* infection. This nodular aspect varied from 32.9% to 85% in children with *H. pyori*, an aspect previously considered to be specific [38].

Domsa et al. found in a recent 5-year retrospective study from northwestern Romania, performed on 82 *H. pylori*-positive children out of 248 patients enrolled, that the endoscopic features such as hyperemia, nodularity pattern and edema were suggestive of pediatric *H. pylori* infection [39].

Regarding the endoscopic lesions, our study revealed that nodular and hyperemic lesions (31.0%) and nodular aspects (22.4%) were the main macroscopic patterns observed in patients with *H. pylori*, similar to previous studies.

Botija et al. analysed the role of adherence in case of treatment failure in paediatric patients receiving antimicrobial susceptibility treatment. They considered it necessary for patients and their families to be properly informed that treatment adherence could play an important role in improving the success rate of eradication [40].

In our study, 9.5% of the patients stopped the treatment mainly because they misunderstood the treatment protocol, and 4.3% had side effects. As a cause of treatment discontinuation, its cost has not been reported, because in Romania, most children’s treatments are free. Instead, some parents said that they did not understand that the treatment was prescribed for 14 days consecutively, or they forgot to administer a dose on some days.

In *H. pylori* infection in adults, experts in Europe, Canada and U.S.A. have developed new recommendations for treatment of *H. pylori* infections that promote quadruple therapy containing bismuth as a first-line empirical therapy. As such, combination capsules containing bismuth, tetracycline and metronidazole have been developed that substantially simplify the treatment with good efficacy and safety. This type of medication is not administered to children, and in Romania there is no such option for adults [41].

Vomiting, nausea, diarrhea, constipation, heartburn, stomach cramps, decreased appetite, headache and dry mouth have been reported as side effects of the therapy. Okuda et al. additionally reported abnormal sensation in the tongue and mouth and tongue irritation [42]. In our study, 4.3% of the patients stopped the treatment because they had nausea or forgot to take the PPI in the morning, so they had severe stomach pain after taking the antibiotics.

One of the major risk factors for failure to eradicate gastric *H. pylori* infection may be poor adherence. Other risk factors that can be considered are high gastric acidity, high bacterial load and lack of sensitivity of the specific *H. pylori* strain to antibiotics. In addition, the lack of awareness of providers of local and national resistance models for specific antibiotics can be considered a risk factor for failure to eradicate the infection [43].

Of the positive patients, 29.3% had persistent symptoms despite treatment. Among the most pronounced symptoms were pain in the epigastric area, diffuse abdominal pain or vomiting. Although these symptoms were present befoare diagnosis, they persisted even after the end of treatment. As such, we can presume that resistant *H. pylori* strains may be the cause of this phenomenon.

In Romania, only a few hospitals have specialized pediatric gastroenterologists and even fewer pediatric endoscopists. Consequently, major difficulties in diagnosing and treating *H. pylori* infection are frequently encountered.

The limitations of this study are mainly the low number of participants within a single geographical area—northeastern Romania—and that it investigates a limited number of cases, as it is a single-centre study. The patient enrolment was conducted partially during the COVID-19 pandemic when the number of upper digestive endoscopies performed routinely or for mild digestive symptoms was limited. In addition, the study was limited to the evaluation of patients with digestive symptoms who presented in a tertiary unit; the general pediatric population was sampled. According to present literature, in Romania, there is a high prevalence of *H. pylori* infection and related disorders [19] and there are limited research studies published on this topic and there is a lack of epidemiological data available. As far as our knowledge goes, this is the first study describing risk factors and treatment compliance in Romanian children.

In addition, due to the COVID-19 pandemic onset during the study, we faced a limitation of the possibility to test antimicrobial resistance. Our hospital was equipped with high-performance PCR equipment but it has been used for a long time only for SARS-CoV-2 testing, and we have been able to use it for non-COVID testing several months after the end of this study period.

Prospective country-wide studies are needed, since Romania is a leading country in empirical antibiotic consumption, and antibiotic resistance of *H. pylori* in our country is still unknown. Our ongoing research includes eradication rates and antibiotic resistance.

## 5. Conclusions

Pediatric *H. pylori* infection has particular epidemiology, clinical features, associated disease, diagnosis and treatment strategies. Romania, with the highest incidence of rural poverty among EU countries, has particular risk factors for pediatric *H. pylori* infection such as living in rural areas, improper sanitary facilities in schools and homes and overcrowded housing. The lack of specialized pediatric gastroenterologists and endoscopists lead to major difficulties in diagnosis and treatment of this infection. Even if treatment compliance is satisfactory, antibiotic resistance may be a leading cause for eradication failure, which is not yet well investigated in Romanian children and which is the subject of our ongoing research.

## Figures and Tables

**Table 1:** Study population characteristics.

	Study Population n (%)	*H. pylori* + n (%)	*H. pylori* − n (%)	*p*-Value *
Total number	185 (100%)	116 (62.7%)	69 (37.3%)	
Age range (years)	6–17 years	6–17 years	6–17 years	0.79
Mean ± SD	M = 13.12; SD = 3.27	M = 13.10, SD = 3.33	M = 13.15, SD = 3.19	
Age groups years, n (%)				
6–9	32 (17.29%)	21 (18.1%)	11 (15.9%)	0.74
10–14	71 (38.39%)	41 (35.3%)	30 (43.5%)	0.17
15–17	82 (44.32%)	54 (46.6%)	28 (40.6%)	0.85
Sex, n (%)				<0.001
Female	126 (68.10%)	76 (65.5%)	50 (72.5%)	0.02
Male	59 (31.90%)	40 (34.5%)	19 (27.5%)	0.09
Place of residence, n (%)				<0.001
Urban	69 (37.30%)	39 (33.6%)	30 (43.5%)	0.33
Rural	116 (62.70%)	77 (66.4%)	39 (56.5%)	0.001
Breastfeeding				<0.001
>6 months	71 (38.38%)	67 (57.8%)	47 (68.1%)	0.07
<6 months	114 (61.62%)	49 (42.2%)	22 (31.9%)	0.02

* Between *H. pylori*-positive and *H. pylori*-negative groups.

**Table 2:** Risk factors associated with *H. pylori* infection.

	Study Population n (%)	*H. pylori* + n (%)	*H. pylori* − n (%)	*p*-Value *
Persons living in a home				<0.001
1 pers/room	51 (27.6%)	26 (22.4%)	25 (36.2%)	1.00
2 pers/room	108 (58.4%)	73 (62.9%)	35 (50.7%)	<0.001
>3 pers/room	26 (14.1%)	17 (14.7%)	9 (13%)	0.16
Type of drinking water				0.30
Bottled water	55 (29.7%)	33 (28.4%)	22 (31.9%)	0.17
Sink water	50 (27.0%)	28 (24.1%)	22 (31.9%)	0.48
Fountain water	80 (43.2%)	55 (47.4%)	25 (36.2%)	0.001
Pets				0.18
Cat	20 (10.8%)	14 (12.1%)	6 (8.7%)	0.11
Dog	15 (8.1%)	11 (9.5%)	4 (5.8%)	0.11
Other	5 (2.7%)	5 (4.3%)	0 (0%)	0.06
None	145 (78.4%)	86 (74.1%)	59 (85.5%)	0.03
Respiratory infection				0.57
1–2/year	141 (76.2%)	90 (77.5%)	51 (73.9%)	<0.001
3–4/year	44 (23.8%)	26 (22.4%)	18 (26.1%)	0.29
>5/year	0 (0%)	0 (0%)	0 (0%)	-
History of antibiotic therapy				0.34
CLR	67 (36.2%)	38 (32.7%)	29 (42.0%)	0.32
AMX	1 (0.5%)	0 (0%)	1 (1.45%)	-
AMC	14 (7.6%)	8 (6.9%)	6 (8.7%)	-
Other	2 (1.08%)	2 (1.72%)	0 (0%)	0.50
Can’t remember	12 (6.5%)	7 (6.03%)	5 (7.2%)	0.77
CLR + AMX	5 (2.7%)	2 (1.72%)	3 (4.3%)	0.50
CLR + AMC	67 (36.2%)	48 (41.3%)	19 (27.5%)	0.001
CLR + MTZ	2 (1.1%)	2 (1.7%)	0 (0%)	0.50
CLR + OTHER	2 (1.1%)	2 (1.7%)	0 (0%)	0.50
AMC + OTHER	1 (0.5%)	1 (0.9%)	0 (0%)	-
CLR + AMX + AMC	3 (1.62%)	2 (1.72%)	1 (1.4%)	1.00
CLR + AMX + OTHER	8 (4.3%)	3 (2.6%)	5 (7.2%)	0.72
CLR + AMX + AMC + MTZ	1 (0.5%)	1 (0.9%)	0 (0%)	-
Familial history of upper digestive pathology				0.32
Yes	31 (16.8%)	17 (14.7%)	14 (20.3%)	0.72
No	154 (83.2%)	99 (85.3%)	55 (79.7%)	<0.001

AMX: amoxicillin; AMC: amoxicillin and clavulanic acid; CLR: clarithromycin; MTZ: metronidazole. * Between *H. pylori*-positive and *H. pylori*-negative groups.

**Table 3:** Clinical presentation symptoms and endoscopic patterns.

	Study Population n (%)	*H. pylori* + n (%)	*H. pylori* − n (%)	*p*-Value *
Clinic				*p* = 0.37
Epigastric pain	105 (56.8%)	66 (56.9%)	39 (56.5%)	0.008
Epigastric + recurrent abdominal pain	3 (1.6%)	2 (1.7%)	1 (1.4%)	0.56
Epigastric pain + vomiting	10 (5.4%)	16 (13.8%)	8 (11.6%)	0.10
Recurrent abdominal pain	27 (14.6%)	18 (15.5%)	9 (13.0%)	0.08
Recurrent abdominal pain + vomiting	24 (12.9%)	3 (2.6%)	7 (10.1%)	0.20
Vomiting	3 (1.6%)	2 (1.7%)	1 (1.4%)	0.56
Refractory anemia	4 (2.2%)	4 (3.4%)	0 (0%)	-
Other symptoms	9 (4.9%)	5 (4.3%)	4 (5.8%)	0.73
Endoscopic pattern				<0.001
Nodular	38 (20.5%)	26 (22.4%)	12 (17.4%)	0.02
Nodular + hyperemic	36 (19.4%)	36 (31.0%)		-
Nodular + hyperemic + snakeskin	2 (1.1%)	1 (0.9%)	1 (1.4%)	1.00
Nodular + erosive	1 (0.5%)	1 (0.9%)	0 (0%)	-
Nodular + snakeskin	1 (0.5%)	0 (0%)	1 (1.4%)	-
Erosive	6 (3.2%)	3 (2.6%)	3 (4.3%)	-
Hyperemic	70 (37.8%)	25 (21.6%)	45 (65.2%)	1.00
Erosive + hyperemic	1 (0.5%)	0 (0%)	1 (1.4%)	0.01
Hyperemic + snakeskin	4 (2.1%)	2 (1,72%)	2 (2.9%)	1.00
Atrophic	26 (14.1%)	22 (19%)	4 (5.8%)	0.001

* Between *H. pylori*-positive and *H. pylori*-negative groups.

**Table 4:** The relationship between chronic inflammation and *H. pylori* density.

	Chronic Inflammation ^1^ (n = 116)			*p*-Value
	Mild (n = 9)	Moderate (n = 75)	Severe (n = 32)	
***H. pylori*^2^, n (%)**				0.002
Mild	5 (55.5%)	41 (54.7%)	9 (28.1%)	
Moderate	1 (11.1%)	25 (33.3%)	17 (53.1%)	
Severe	3 (33.4%)	9 (12.0%)	6 (18.8%)	

^1^ Shows the intensity of lymphocytes and plasma cells in lamina propria according to the Sydney System. ^2^ Refers to the density of *H. pylori* in gastric mucosa.

**Table 5:** Eradication scheme and therapy compliance in *H. pylori*-positive patients.

	*H. pylori* + (n = 116)
Treatment	
PPI + AMX + CLR *	49 (42.2%)
PPI + AMX + MTZ	62 (53.4%)
PPI + AMC + MTZ	5 (4.3%)
Duration of treatment	
14 days	116 (100.0%)
Discontinuation of treatment	
Yes	16 (13.8%)
No	100 (86.2%)
Cause	
Treatment cost	0 (0%)
Side effects	5 (4.3%)
Misunderstanding of the treatment protocol	11 (9.5%)
Side effects	
Yes	34 (29.3%)
No	82 (70.6%)
Persistence of symptoms	
Yes	32 (27.5%)
No	84 (72.4%)

* PPI: proton pomp inhibitor; AMX: amoxicillin; CLR: clarithromycin; MTZ: metronidazole.

## Data Availability

Not applicable.
